# Antimicrobial resistance status of *Enterococcus* from Australian cattle populations at slaughter

**DOI:** 10.1371/journal.pone.0177728

**Published:** 2017-05-18

**Authors:** Robert S. Barlow, Kate E. McMillan, Lesley L. Duffy, Narelle Fegan, David Jordan, Glen E. Mellor

**Affiliations:** 1 CSIRO Agriculture and Food, Coopers Plains, Queensland, Australia; 2 CSIRO Agriculture and Food, Werribee, Victoria, Australia; 3 New South Wales Department of Primary Industries, Wollongbar, New South Wales, Australia; Universitatsklinikum Munster, GERMANY

## Abstract

Antimicrobial agents are used in cattle production systems for the prevention and control of bacterial associated diseases. A consequence of their use is the potential development of antimicrobial resistance (AMR). *Enterococcus faecium* and *Enterococcus faecalis* that are resistant to antimicrobials are of increased concern to public health officials throughout the world as they may compromise the ability of various treatment regimens to control disease and infection in human medicine. Australia is a major exporter of beef; however it does not have an ongoing surveillance system for AMR in cattle or foods derived from these animals. This study examined 910 beef cattle, 290 dairy cattle and 300 veal calf faecal samples collected at slaughter for the presence of enterococci. *Enterococcus* were isolated from 805 (88.5%) beef cattle faeces, 244 (84.1%) dairy cattle faeces and 247 (82.3%) veal calf faeces with a total of 800 enterococci subsequently selected for AMR testing. The results of AMR testing identified high levels of resistance to antimicrobials that are not critically or highly important to human medicine with resistance to flavomycin (80.2%) and lincomycin (85.4–94.2%) routinely observed. Conversely, resistance to antibiotics considered critically or highly important to human medicine such as tigecycline, daptomycin, vancomycin and linezolid was not present in this study. There is minimal evidence that Australian cattle production practices are responsible for disproportionate contributions to AMR development and in general resistance to antimicrobials of critical and high importance in human medicine was low regardless of the isolate source. The low level of antimicrobial resistance in *Enterococcus* from Australian cattle is likely to result from comprehensive controls around the use of antimicrobials in food-production animals in Australia. Nevertheless, continued monitoring of the effects of all antimicrobial use is required to support Australia’s reputation as a supplier of safe and healthy food.

## Introduction

Australia produces approximately 4% of the world’s beef supply yet exports more than 70% of production making it the world’s largest beef exporter in 2015 [1]. Antimicrobial agents are used in cattle production systems for the prevention and control of bacterial associated diseases. As many classes of antimicrobials are approved for and may be used in cattle production systems there is the potential for antimicrobial resistance (AMR) to develop in bacteria, including zoonotic pathogens which can be transferred to the human population via the food chain or by direct exposure to animals [2, 3]. Novel resistance phenotypes continue to emerge in zoonotic foodborne pathogens and commensal bacteria isolated from food production animals [4, 5]. In particular, *Enterococcus faecalis* and *Enterococcus faecium* have become of increasing importance over recent decades because of life-threatening hospital-acquired infections [6]. Consequently, understanding, assessing and mitigating the risks of non-human use of antimicrobials on human health outcomes remains a high priority. The World Health Organisation (WHO) has developed and maintains criteria and ranks antimicrobials based on their importance to human medicine [7]. Such information will help regulators and stakeholders identify appropriate antimicrobials for use in food animal production systems [8].

A number of countries have established AMR surveillance programs in place. Whilst the main focus of these programs is AMR in bacteria from humans, there is considerable and increasing emphasis on assessing AMR in bacteria from animals during production and from foods at retail. Multi-focus surveillance programs enable trends in AMR development to be further evaluated with respect to production practices and animal type and are particularly useful in addressing concerns from regulators about the overall impact of antimicrobial use. Countries without sophisticated multi-focus surveillance programs instead rely on relatively short-term intensive surveys to achieve the same result. The aim of this study was to conduct a short-term surveillance program to determine the prevalence and AMR status of *Enterococcus* isolates from Australian cattle populations.

## Materials and methods

### Sample collection

Faecal samples were collected from Australian cattle at slaughter as previously described [9]. Briefly, samples were collected from three animal groups: beef cattle, dairy cattle, and veal calves. Animals were defined as veal if their carcass weight was no more than 150kg. A total of 910, 290 and 300 samples were collected from beef cattle, dairy cattle and veal calves, respectively. Samples were collected across two sampling windows with sampling occurring once per abattoir in each window. Abattoirs were expected to collect up to a maximum of 40 samples per sampling day therefore all samples were expected to be collected a minimum of 12 minutes apart. Faecal samples were collected post-evisceration by cutting the intestine 15–30 cm from the rectal end and squeezing at least 40 g of material into a sterile jar. Samples were kept chilled and returned to the laboratory by overnight courier for processing.

### Isolation of *Enterococcus*

The presence of *Enterococcus* was determined by enriching 1 g of faeces in 10 ml of BBL Enterococcosel Broth (BD, Maryland, USA) for 18–24 h at 35 ± 2°C. Enriched broths were then plated onto BBL Enterococcosel Agar (BD) and incubated for 18–24 h at 35 ± 2°C. Translucent colonies with brownish-black to black zones were then streaked onto Sheep Blood Agar (SBA) and incubated for 18–24 h at 35 ± 2°C. Isolates were confirmed as *Enterococcus* spp. by PCR [10]. A species specific PCR was then used to identify *E*. *faecalis* and *E*. *faecium* strains [11]. Further speciation was not performed and the remaining isolates were labelled *Enterococcus spp*.

### Phenotypic detection of antimicrobial resistance

The AMR phenotype of isolates was initially determined using the broth microdilution method and the Sensititre apparatus. A total of 800 *Enterococcus* isolates comprising all *E*. *faecium* and *E*. *faecalis* and a selection of *Enterococcus spp*. Isolates from across the three animal groups were selected for AMR testing. Custom susceptibility panels for *Enterococcus* (AUSVP2; TREK Diagnostic Systems, UK) were used to test all isolates. The dilution ranges and breakpoints for each antimicrobial are shown in Table 1. Interpretation of the MIC values was based on CLSI interpretive criteria when available; otherwise EUCAST and NARMS values were used. Isolates that exceeded the MIC value of the susceptible breakpoint were reported as non-susceptible. *E*. *faecalis* ATCC 29212 was used as the control strain.

**Table 1:** Dilution ranges and breakpoints for antimicrobial susceptibility testing.

Antimicrobial	Range	Breakpoint^*^
Ampicillin	0.5–16	≥16
Chloramphenicol	2–32	≥32
Daptomycin	0.125–4	≥8
Erythromycin	0.25–8	≥8
Flavomycin	1–32	≥32
Gentamicin	32–1024	≥512
Kanamycin	128–1024	≥1024
Lincomycin	1–32	≥8
Linezolid	0.5–8	≥8
Penicillin	0.5–16	≥16
Streptomycin	256–1024	≥1024
Teicoplanin	0.125–4	2
Tetracycline	2–16	≥16
Tigecycline	0.016–0.5	≥0.5
Vancomycin	0.25–32	≥32
Virginiamycin	1–32	>8

^*^ CLSI breakpoints were adopted for ampicillin, daptomycin, erythromycin, linezolid, penicillin and vancomycin. NARMS breakpoints were used for chloramphenicol, flavomycin, gentamicin, kanamycin, lincomycin, streptomycin, tetracycline, tigecycline with EUCAST breakpoints used for teicoplanin and virginiamycin.

Isolates that demonstrated resistance to antimicrobials (e.g daptomycin and tigecycline) of critical or high importance to human medicine in the Sensititre testing process were further evaluated using M.I.C. Evaluator strips (Oxoid, UK). Susceptibility testing was conducted as per the manufacturer’s recommendations with each isolate suspended in cation adjusted Mueller-Hinton broth at 0.5 MacFarland standard. Each isolate was subsequently spread plated onto Mueller-Hinton agar and overlaid with the appropriate MIC Evaluator strip. The MIC or zone of clearance was measured after 24 hours incubation at 37°C.

### Genotypic detection of antimicrobial resistance

Isolates that demonstrated resistance to daptomycin and tigecycline were tested for the presence of AMR genes or SNPs that have previously been shown to be associated with resistance to the aforementioned antimicrobials [12–14]. The primers, cycling conditions and expected product sizes are shown in Table 2. Detection of SNPs in *liaR*, *liaS* and *rpsJ* was conducted by Sanger sequencing of PCR products (AGRF, Brisbane) and subsequent analysis in Vector NTi (Life Technologies, Australia).

**Table 2:** Primers, cycling conditions and expected product sizes of *Enterococcus* AMR gene PCRs.

Resistance to:	Oligo (5’–3’)	Cycling conditions	Products size (bp)	Reference
Daptomycin	liaR-F:GGTCCGATCATCCACATCTA liaR-R:CCGTTTAGGCGTTTCATCAT	30s 94°C, 30s 60°C, 30s 72°C x 30	553	This study
	liaS-F:AAAGTCATTGGTGGGGAGAA liaS-R:GACTGGGAAGCGTTGATGAT	30s 94°C, 30s 60°C, 30s 72°C x 30	526	
Tigecycline	rpsJ-F:AGAGGTTGCGACACGCCCGG rpsJ-R:TCTACAACAGTTACTGGAAT	30s 94°C, 30s 60°C, 30s 72°C x 30	525	[13]

## Results

### Prevalence and identity

In total, 1500 faecal samples comprising 910 beef cattle faeces, 290 dairy cattle faeces and 300 veal calf faeces were tested for the presence of *Enterococcus*. *Enterococcus* were isolated from 805 (88.5%) beef cattle faeces, 244 (84.1%) dairy cattle faeces and 247 (82.3%) veal calf faeces. Species specific PCR determined that 96 (6.4%) of faecal samples yielded *E*. *faecalis* and 120 (8.0%) yielded *E*. *faecium*. Veal samples (14.3%) were significantly (p = 0.05) more likely to contain *E*. *faecalis* than dairy (3.1%) or beef (4.8%) samples. No significant differences in prevalence were observed between the three animal groups for *E*. *faecium*.

### Antimicrobial susceptibility testing

#### Sensititre evaluation

A total of 800 *Enterococcus* isolates comprising 96 *E*. *faecalis*, 120 *E*. *faecium*, and 584 *Enterococcus spp*. were submitted for AMR analysis using the Sensititre test system. The distribution of MICs for each antimicrobial and species group is shown in Table 3. Breakpoints are not available for unspeciated *Enterococcus* isolates and therefore resistance data is only shown for *E*. *faecium* and *E*. *faecalis*. Streptogramin MIC values for *E*. *faecalis* are not presented as this species is intrinsically resistant. Similarly, flavomycin MIC values for *E*. *faecium* are not shown as they are inherently nonsusceptible. Irrespective of animal group and species, resistance to flavomycin (77.3–88.9%) and lincomycin (77.8–100.0%) was common. There was a strong association between daptomycin resistant *E*. *faecalis* and veal calves, however this was not considered to be statistically significant (p = 0.05). Resistance to tetracycline (2.3–13.0%) and erythromycin (0.0–13.6%) were observed in the majority of the three animal groups except for erythromycin resistance in *E*. *faecium* from veal calves. Furthermore, tigecycline resistance was only observed in *E*. *faecium* and *E*. *faecalis* from grass-fed animals, and whilst tetracycline resistance in *E*. *faecalis* was more common in grain-fed isolates, the opposite relationship existed in *E*. *faecium* with tetracycline resistance only detected in isolates from grass-fed animals.

**Table 3:** Distribution of MICs and occurrence of resistance among *Enterococcus* isolates using the Sensititre test system.

Class	Antimicrobial	Species	N =	% Resistant	95% CI	Antimicrobial concentration (μg/ml)																	
						0.015	0.03	0.06	0.12	0.25	0.5	1	2	4	8	16	32	64	128	256	512	1024	>1024
**Aminoglycoside**	**Gentamicin**	*Enterococcus faecalis*	96	0.0	0.00–3.77												100.0						
		*Enterococcus faecium*	120	0.0	0.00–3.03												99.2	0.8					
		*Enterococcus spp*	584	NA	NA												99.7	0.3					
	**Kanamycin**	*Enterococcus faecalis*	96	1.0	0.03–5.67														92.7	5.2	1.0	1.0	
		*Enterococcus faecium*	120	0.8	0.02–4.56														99.2			0.8	
		*Enterococcus spp*	584	NA	NA														95.0	3.8	0.3	0.3	0.5
	**Streptomycin**	*Enterococcus faecalis*	96	1.0	0.03–5.67															99.0			1.0
		*Enterococcus faecium*	120	0.0	0.00–3.03															100.0			
		*Enterococcus spp*	584	NA	NA															99.1			0.9
**Glycopeptides**	**Teicoplanin**	*Enterococcus faecalis*	96	0.0	0.00–3.77				30.2	46.9	17.7	5.2											
		*Enterococcus faecium*	120	0.0	0.00–3.03				42.5	40.0	14.2	3.3											
		*Enterococcus spp*	584	NA	NA				33.2	41.1	19.7	6.0											
	**Vancomycin**	*Enterococcus faecalis*	96	0.0	0.00–3.77					1.0	40.6	34.4	11.5	10.4	2.1								
		*Enterococcus faecium*	120	0.0	0.00–3.03						46.7	22.5	15.0	15.0	0.8								
		*Enterococcus spp*	584	NA	NA					0.5	41.6	28.3	13.4	12.3	3.9								
**Glycylcycline**	**Tigecycline**	*Enterococcus faecalis*	96	2.1	0.25–7.32	2.1	45.8	41.7	3.1	5.2	2.1												
		*Enterococcus faecium*	120	2.5	0.52–7.13		43.3	44.2	5.8	4.2	2.5												
		*Enterococcus spp*	584	NA	NA	2.1	45.0	37.7	8.0	4.3	2.7	0.2											
**Lincosamide**	**Lincomycin**	*Enterococcus faecalis*	96	85.4	76.74–91.79							10.4	1.0	3.1	8.3	25.0	34.4	17.7					
		*Enterococcus faecium*	120	94.2	88.35–97.62							4.2	0.8	0.8	2.5	27.5	50.0	14.2					
		*Enterococcus spp*	584	NA	NA							12.3	1.9	2.1	6.2	30.7	37.5	9.4					
**Lipopeptide**	**Daptomycin**	*Enterococcus faecalis*	96	9.4	4.38–17.05								75.0	15.6	9.4								
		*Enterococcus faecium*	120	2.5	0.52–7.13							5.8	45.0	46.7	2.5								
		*Enterococcus spp*	584	NA	NA				0.2		3.3	24.8	40.6	24.5	6.7								
**Macrolide**	**Erythromycin**	*Enterococcus faecalis*	96	10.4	5.11–18.32					33.3	15.6	14.6	20.8	5.2	1.0	9.4							
		*Enterococcus faecium*	120	8.3	4.07–14.79					53.3	13.3	12.5	9.2	3.3	4.2	4.2							
		*Enterococcus spp*	584	NA	NA					45.9	13.2	11.0	17.6	6.3	1.2	4.8							
**Oxazolidinones**	**Linezolid**	*Enterococcus faecalis*	96	0.0	0.00–3.77							6.3	87.5	6.3									
		*Enterococcus faecium*	120	0.0	0.00–3.03							5.8	87.5	6.7									
		*Enterococcus spp*	584	NA	NA						0.3	5.0	88.2	6.5									
**Penicillins**	**Ampicillin**	*Enterococcus faecalis*	96	0.0	0.00–3.77						44.8	52.1	3.1										
		*Enterococcus faecium*	120	0.0	0.00–3.03						30.0	60.0	10.0										
		*Enterococcus spp*	584	NA	NA						45.9	29.5	22.3	2.4									
	**Penicillin**	*Enterococcus faecalis*	96	0.0	0.00–3.77						13.5	28.1	26.0	31.3	1.0								
		*Enterococcus faecium*	120	0.0	0.00–3.03						12.5	34.2	30.8	20.8	1.7								
		*Enterococcus spp*	584	NA	NA						19.3	27.1	22.8	21.2	9.2	0.2	0.2						
**Phenicol**	**Chloramphenicol**	*Enterococcus faecalis*	96	0.0	0.00–3.77									17.7	82.3								
		*Enterococcus faecium*	120	0.0	0.00–3.03								1.7	51.7	40.8	5.8							
		*Enterococcus spp*	584	NA	NA								0.3	38.4	59.4	1.9							
**Phosphoglycolipid**	**Flavomycin#**	*Enterococcus faecalis*	96	80.2	70.83–87.64							15.6	1.0		2.1	1.0	2.1	78.1					
		*Enterococcus faecium*	NA	NA	NA																		
		*Enterococcus spp*	584	NA	NA							11.8	1.0	0.7	1.0	0.7	2.1	82.7					
**Streptogramins**	Virginiamycin*	*Enterococcus faecalis*	NA	NA	NA																		
		*Enterococcus faecium*	120	0.0	0.00–3.03							85.0	5.8	9.2									
		*Enterococcus spp*	584	NA	NA							86.6	6.8	6.0	0.2	0.3							
**Tetracycline**	**Tetracycline**	*Enterococcus faecalis*	96	7.3	2.98–14.45								86.5	2.1	4.2	3.1	4.2						
		*Enterococcus faecium*	120	11.7	6.53–18.80								78.3	8.3	1.7	1.7	10.0						
		*Enterococcus spp*	584	NA	NA								83.2	3.9	2.1	1.9	8.9						

^*^ *Enterococcus faecalis* isolates are intrinsically resistant to streptogramins

^#^ *Enterococcus faecium* isolates are inherently nonsuceptible to flavomycin.

Solid vertical lines indicate breakpoints for resistance. The white fields indicate the dilution range tested for each antimicrobial. Values in the shaded area indicate MIC values greater than the highest concentration tested.

#### Additional phenotypic AMR testing

Initial evaluation of AMR in the *Enterococcus* isolates identified resistance to antimicrobials of human clinical significance. In particular, resistance to daptomycin and tigecycline was noted. Resistance to these antimicrobials was higher than anticipated and therefore was further investigated. Testing of daptomycin and tigecycline was completed in duplicate on each of the isolates that had previously demonstrated resistance to these antimicrobials in the Sensititre system, using the M.I.C. Evaluator system. MICs for tigecycline were all below the clinical breakpoint and therefore all isolates should be considered susceptible to tigecycline. The three *E*. *faecium* and two *E*. *faecalis* isolates all had an MIC of 0.12 μg/mL and are consistent with wild-type strains. None of the *Enterococcus spp*. isolates had MICs greater than the clinical breakpoint used for *E*. *faecium* and *E*. *faecalis*. Similarly, the three *E*. *faecium* and nine *E*. *faecalis* isolates previously identified as resistant to daptomycin all had MICs below the clinical breakpoint on re-testing. The *E*. *faecium* isolates all had MICs of 2 μg/mL whereas the *E*. *faecalis* isolates ranged from 0.25 to 2 μg/mL. One *Enterococcus* spp. isolate had an elevated MIC of 8 μg/mL, however the MICs of all remaining isolates were below the clinical breakpoint.

#### Genotypic investigation of AMR

In an attempt to corroborate the findings of the additional daptomycin and tigecycline AMR testing, all isolates including those not identified as *E*. *faecalis* or *E*. *faecium*, exhibiting MICs greater than the clinical breakpoints for daptomycin or tigecycline were tested by PCR for a range of genetic markers known to be associated with resistance to these antimicrobials. In total, 42 daptomycin resistant isolates and 22 tigecycline resistant isolates were tested further. For tigecycline resistant isolates, fragments of *rpsJ* were amplified, sequenced and analysed for a SNP that encodes a predicted amino acid change of Asp60 to Tyr. Fragments of *rpsJ* were amplified from all three *E*. *faecium* isolates and from 14 (82.4%) of 17 *Enterococcus* spp. isolates. Fragments of *rpsJ* were not amplified in either of the two *E*. *faecalis* isolates. Analysis of the 17 *rpsJ* fragments determined that none of the isolates harboured the SNP that has been shown to be associated with reduced susceptibility to tigecycline. Daptomycin resistant isolates were tested for the presence of SNPs in *liaR* and *liaS*. PCR products for *liaR* or *liaS* were generated from eight (88.9%) of nine *E*. *faecalis* isolates and all three *E*. *faecium* isolates but was found in only two (6.7%) of 30 *Enterococcus* spp. isolates. *E*. *faecalis* strains were most likely to harbour *liaR* on its own whereas *E*. *faecium* were more likely to harbour *liaS*. One *E*. *faecalis* and one *E*. *faecium* isolate were shown to contain both *liaR* and *liaS*. Sequencing of the PCR fragments determined that none of the isolates possessed the Thr120 to Ala SNP in *liaR* or the Trp73 to Cys SNP in *liaS*.

#### Re-categorisation of daptomycin and tigecycline results

**Fig 1:**
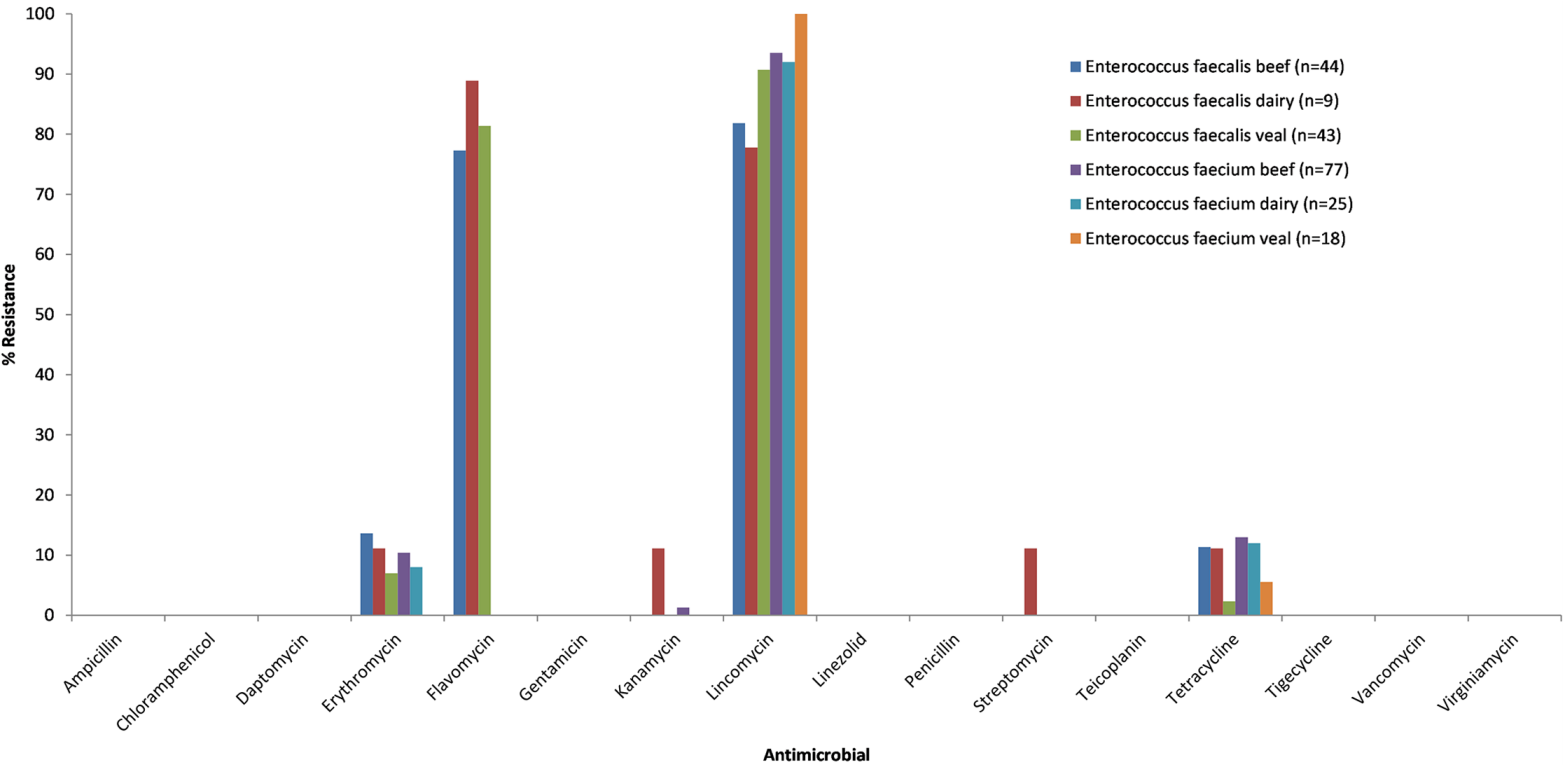
Prevalence of AMR in *Enterococcus faecalis and faecium* isolates from beef cattle, dairy cattle and veal calf faecal samples.

The inability to reproduce the findings of the primary phenotypic antimicrobial testing conducted using the Sensititre test system with custom AMR plates and the absence of the identification of AMR-linked genetic markers suggests that the original phenotypic assessment for resistance to daptomycin and tigecycline is incorrect and likely comprised of major errors. The major error rates for *E*. *faecalis* isolates against daptomycin and tigecycline were 9.4% and 2.1%, respectively. Whilst the major error rates for *E*. *faecium* isolates against daptomycin and tigecycline were low at 2.5% for both. The acceptance of major errors in the assessment of the three antimicrobials results in the re-categorisation of the results for the *E*. *faecalis and E*. *faecium* isolates in question. The revised results are shown in Table 4 and the distribution of AMR in *E*. *faecalis* and *E*. *faecium* from the three animal groups shown in Fig 1.

**Table 4:** Revised MICs and occurrence of resistance among *Enterococcus* isolates following additional phenotypic and genotypic assessment.

Class	Antimicrobial	Species	N =	% Resistant	95% CI	Antimicrobial concentration (μg/ml)										
						0.015	0.03	0.06	0.12	0.25	0.5	1	2	4	8	16
**Glycylcycline**	**Tigecycline**	*Enterococcus faecalis*	96	0.0	0.00–3.77	2.1	45.8	41.7	5.2	5.2						
		*Enterococcus faecium*	120	0.0	0.00–3.03		43.3	44.2	5.8	4.2						
		*Enterococcus spp*	584	NA	NA	2.1	45.0	37.7	8.0	4.3						
**Lipopeptide**	**Daptomycin**	*Enterococcus faecalis*	96	9.4	0.00–3.77					2.1	4.2	2.1	76.0	15.6		
		*Enterococcus faecium*	120	2.5	0.00–3.03							5.8	47.5	46.7		
		*Enterococcus spp*	584	NA	NA				0.2	0.7	5.3	25.5	40.9	27.2	0.2	

Solid vertical lines indicate breakpoints for resistance. The white fields indicate the dilution range tested for each antimicrobial.

#### Antimicrobial resistance profiles

Resistance to three or more classes of antimicrobial, which we defined as multidrug resistance (MDR), was observed in 18 (8.3%) of all *E*. *faecium* and *E*. *faecalis* isolates. Table 5 shows the resistance profiles for each *E*. *faecium* and *E*. *faecalis*. MDR was observed in six (5.0%) *E*. *faecium* and 12 (12.5%) *E*. *faecalis* isolates. Resistance to four or more antimicrobial classes was less commonly observed with only four (3.3%) *E*. *faecium* isolates falling into this category. Antimicrobial resistance profiles of *E*. *faecium* were dominated by resistance to lincomycin (78.3%). FLV-LIN was the most common resistance profile associated with *E*. *faecalis* isolates with 53 (55.2%) of 96 isolates harbouring this combination. The main MDR profiles for *E*. *faecium* and *E*. *faecalis* were ERY-LIN-TET (3.3%) and ERY-FLV-LIN (5.2%), respectively.

**Table 5:** Antimicrobial resistance profiles of *Enterococcus faecium* and *faecalis* isolates from beef cattle, dairy cattle and veal calf faecal samples.

Antimicrobial resistance profile^*^	*E*. *faecium* (N = 120)	*E*. *faecalis* (N = 96)
ALL SENSITIVE	6	1
FLV		13
LIN	95	17
TET	1	
ERY LIN	5	
FLV LIN		53
LIN TET	8	
ERY FLV LIN		5
ERY LIN TET	4	1
ERY KAN LIN	1	
FLV LIN TET		2
ERY FLV LIN TET		3
ERY FLV KAN LIN STR TET		1

* FLV–flavomycin, LIN–lincomycin, TET–tetracycline, ERY–erythromycin, KAN–kanamycin, STR—streptomycin

## Discussion

Bacteria that are resistant to antimicrobials are of increased concern to public health officials throughout the world as they may compromise the ability of treatment regimens to address disease and infection in humans. Knowledge and understanding of both the types of AMR present in food production animals and the type of antimicrobials being used is key to determining the risk that AMR bacteria in the food chain pose to human health. Australia currently does not have a nationally coordinated program for the ongoing surveillance and analysis of AMR bacteria in animals or bacteria in food derived from animals. Consequently it relies heavily on testing of human and animal clinical isolates as well as infrequent surveys of isolates from animals or from food of animal origin to understand AMR development and trends.

Enterococci are ubiquitous bacteria that demonstrate intrinsic resistance to a number of first-line antimicrobial agents and have also demonstrated capacity to rapidly acquire resistance to antimicrobials including quinolones, macrolides, tetracyclines, streptogramins and glycopeptides [15]. They are also frequently associated with mobile genetic elements harbouring AMR genes and have the potential for resistance to virtually all antimicrobials of importance to human medicine [16]. The importance of enterococci as the third most commonly isolated nosocomial pathogen [17] and the clear relationship between exposure to parental antimicrobials and the development of resistance [18] warrants their ongoing inclusion in any human, animal or food AMR surveillance program. *E*. *faecalis* and *E*. *faecium* were recovered from 6.4% and 8.0% of samples in this survey and although they are the two enterococcal species most associated with human infections, monitoring of additional species of enterococci other than *E*. *faecalis* and *E*. *faecium* is useful as it may provide insights to trends of MIC’s which may be of concern to the more clinically relevant species. From a human clinical perspective, resistance in *E*. *faecalis* and *E*. *faecium* to ampicillin, vancomycin, linezolid, daptomycin and tigecycline are the key concerns. Resistance to other older antimicrobials such as lincomycin, flavomycin, tetracycline and erythromycin are seldom considered, as either resistance is common or the antimicrobials are seldom used in human medicine [18]. The findings of this study reinforce this segregation of concern with increased levels of resistance to lincomycin, flavomycin (*E*. *faecalis* only), tetracycline and erythromycin observed in *E*. *faecium* and *E*. *faecalis* isolates from all animal groups. Furthermore, whilst the highest levels of resistance in this study were to lincomycin and flavomycin, these results are consistent with published studies on enterococci from cattle [19] and likely stem from intrinsic resistance as opposed to the development of resistance resulting from widespread use of these antimicrobials.

Conversely, resistance to antimicrobials of critical and high importance to human medicine is of much greater concern to the ongoing treatment strategies for enterococcal infections. Resistance to ampicillin, linezolid and vancomycin was not observed in this study in any *E*. *faecium* or *E*. *faecalis* isolates. This is significant as ampicillin remains the preferred therapy for uncomplicated enterococcal infections. Similarly, the absence of vancomycin resistant enterococcus assists in maintaining optimal treatment options. Resistance to daptomycin and tigecycline initially observed with the Sensititre system could not be confirmed using gradient diffusion techniques. Publications detailing genes conferring resistance to these antimicrobials in *Enterococcus* isolates are extremely limited, however whole genome analysis of strains demonstrating reduced susceptibility have identified a number of single nucleotide polymorphisms (SNPs) present in those isolates when compared with wild-type populations [12–14, 20]. Importantly, recent studies have demonstrated that *rpsJ* mutations may also be present in tigecycline susceptible isolates and perhaps represent just a small component of a complex interplay between numerous resistance mechanisms required to overcome the selective pressure of tigecycline [21]. Investigation of the SNPs in the *liaFSR* regulon and *rpsJ* determined that the isolates in this study share sequence homology with wild-type isolates and do not contain these known AMR associated mutations. When combined with the agar dilution results the original Sensititre test results are believed to be incorrect and the overall data set has been modified to reflect these findings. As a consequence, this study reports that resistance to the critical or high importance antimicrobials linezolid, daptomycin, tigecycline and vancomycin was not detected in enterococcal isolates from Australian cattle regardless of the production system.

The generation of discordant AMR results after testing with multiple phenotypic test systems is concerning though not confined to this study alone. Several studies have detailed discrepancies in essential agreement and categorical agreement between test systems when single antimicrobial / bacteria combinations are considered. The United States Food and Drug Administration will approve the marketing of AMR tests system provided that very major errors (false-negatives) and major errors (false-positives) do not exceed 1.5% and 3% respectively and essential MIC agreement within one doubling MIC dilution of >90% occurs between the test system and the reference CLSI method [22]. This study has identified major errors with daptomycin and tigecycline, however only the combination of daptomycin with *E*. *faecalis* strains exceed the allowable 3% major error rate. The *in vitro* evaluation of daptomycin and tigecycline resistance has been shown to be highly dependent on the culture conditions used and may provide an explanation for the higher than acceptable major error rate observed in this study [23–25].

Overall, the results corroborate previous Australian based animal and retail food surveys that have shown a low level of AMR, relatively small proportions of MDR and most importantly the maintenance of susceptibility to most antimicrobials of critical and high importance to human health [9, 26–29]. Importantly, it would appear that the production practices in Australian cattle populations are not generating pools of resistance that are likely to result in the inability to treat human infections caused by enterococci. Nevertheless, it is necessary to maintain strict guidelines and controls around the use of antimicrobials in food-production animals in Australia and monitoring the effects of all antimicrobial use is required to support Australia’s reputation as a supplier of safe and healthy food.

## Supporting information

S1 File *Enterococcus* AMR dataset. (XLSX)
